# Delayed Astrogliosis Associated with Reduced M1 Microglia Activation in Matrix Metalloproteinase 12 Knockout Mice during Theiler’s Murine Encephalomyelitis

**DOI:** 10.3390/ijms20071702

**Published:** 2019-04-05

**Authors:** Florian Hansmann, Ning Zhang, Vanessa Herder, Eva Leitzen, Wolfgang Baumgärtner

**Affiliations:** Department of Pathology, University of Veterinary Medicine Hannover; Center for Systems Neuroscience 30559 Hannover, Germany; Florian.Hansmann@tiho-hannover.de (F.H.); zhangning1111@126.com (N.Z.); Vanessa.Herder@tiho-hannover.de (V.H.); Eva.Leitzen@tiho-hannover.de (E.L.)

**Keywords:** astrogliosis, chemokine receptor 2, knockout mice, matrix metalloproteinases, microglia phenotype, M1, M2, Theiler’s murine encephalomyelitis

## Abstract

Theiler’s murine encephalomyelitis (TME) represents a versatile animal model for studying the pathogenesis of demyelinating diseases such as multiple sclerosis. Hallmarks of TME are demyelination, astrogliosis, as well as inflammation. Previous studies showed that matrix metalloproteinase 12 knockout (*Mmp12^−/−^*) mice display an ameliorated clinical course associated with reduced demyelination. The present study aims to elucidate the impact of MMP12 deficiency in TME with special emphasis on astrogliosis, macrophages infiltrating the central nervous system (CNS), and the phenotype of microglia/macrophages (M1 or M2). SJL wild-type and *Mmp12^−/−^* mice were infected with TME virus (TMEV) or vehicle (mock) and euthanized at 28 and 98 days post infection (dpi). Immunohistochemistry or immunofluorescence of cervical and thoracic spinal cord for detecting glial fibrillary acidic protein (GFAP), ionized calcium-binding adaptor molecule 1 (Iba1), chemokine receptor 2 (CCR2), CD107b, CD16/32, and arginase I was performed and quantitatively evaluated. Statistical analyses included the Kruskal–Wallis test followed by Mann–Whitney *U* post hoc tests. TMEV-infected *Mmp12^−/−^* mice showed transiently reduced astrogliosis in association with a strong trend (*p* = 0.051) for a reduced density of activated/reactive microglia/macrophages compared with wild-type mice at 28 dpi. As astrocytes are an important source of cytokine production, including proinflammatory cytokines triggering or activating phagocytes, the origin of intralesional microglia/macrophages as well as their phenotype were determined. Only few phagocytes in wild-type and *Mmp12^−/−^* mice expressed CCR2, indicating that the majority of phagocytes are represented by microglia. In parallel to the reduced density of activated/reactive microglia at 98 dpi, TMEV-infected *Mmp12^−/−^* showed a trend (*p* = 0.073) for a reduced density of M1 (CD16/32- and CD107b-positive) microglia, while no difference regarding the density of M2 (arginase I- and CD107b-positive) cells was observed. However, a dominance of M1 cells was detected in the spinal cord of TMEV-infected mice at all time points. Reduced astrogliosis in *Mmp12^−/−^* mice was associated with a reduced density of activated/reactive microglia and a trend for a reduced density of M1 cells. This indicates that MMP12 plays an important role in microglia activation, polarization, and migration as well as astrogliosis and microglia/astrocyte interaction.

## 1. Introduction

Theiler’s murine encephalomyelitis (TME) represents a well-established animal model for the primary and secondary progressive form of multiple sclerosis (MS; [1,2,3,4,5]). TME virus (TMEV) infection of susceptible mice (such as SJL mice) with low neurovirulent TMEV strains (such as BeAn) results in a biphasic disease course consisting of an acute polioencephalitis followed by a chronic, progressive leukomyelitis with demyelination, astrogliosis, and virus persistence in the spinal cord [4,6,7,8]. Essential hallmarks of TME are progressive gait disabilities, including ataxia, progressing to spastic paresis; progressive demyelination based on a delayed-type hypersensitivity reaction, initially targeting viral antigens and later myelin components; astrogliosis; and axonal degeneration and loss [7,9,10,11,12,13]. Spinal cord inflammation starts approximately four weeks postinfection and progressively increases over time in parallel with astrogliosis and deposition of various extracellular matrix proteins [7]. The inflammatory reaction predominantly consists of microglia/macrophages and lymphocytes leading to axonal degeneration and demyelination [7,13]. TMEV has been detected in numerous central nervous system (CNS) cell types, including neurons, oligodendrocytes, microglia, and astrocytes, depending on the respective stage postinfection [14,15,16,17,18,19]. Degenerative changes predominate in infected neurons and oligodendrocytes, leading to axonal loss and demyelination, whereas infected microglia/macrophages and astrocytes secrete numerous cytokines and chemokines including interferon (IFN) α/β, IL-1, IL-12, tumor necrosis factor (TNF), IL-10, and monocyte chemotactic protein 1, thereby inducing an antiviral immune response [4,6,20,21,22,23,24,25,26,27,28,29]. In this context, matrix metalloproteinases (MMPs) play an important role since they are involved in physiological processes such as angiogenesis, axonal growth, myelinogenesis, and neurogenesis as well as in pathological conditions such as inflammation, demyelination, and blood-brain barrier breakdown [30]. In addition, MMP12 has been shown to cleave a wide range of myelin and extracellular matrix molecules in vitro, and the upregulation of MMP12 has been demonstrated in MS, TME, and experimental autoimmune encephalomyelitis [6]. In addition, previous studies showed an intralesional expression of MMP12 in astrocytes as well as microglia/macrophages [31]. Furthermore, infection of matrix-metalloproteinase-12-deficient (*Mmp12^−/−^*) mice with the BeAn strain of TMEV resulted in an improved clinical course, reduced demyelination, as well as a reduced intralesional number of microglia/macrophages in the spinal cord [6]. These observations suggest that MMP12 has an essential impact on the development and/or progression of demyelinating diseases of the CNS.

Therefore, it was hypothesized that a lack of MMP12 in TMEV-infected mice is responsible for the reduced number of microglia/macrophages and plays a role in microglia/macrophage activation as well as polarization. Microglia/macrophages can be classified into different categories according to their cytokine expression pattern [32]. “Classically activated” macrophages, also called M1 cells, are involved in T helper cell type 1 (Th1) responses, mainly defense orientated, and release proinflammatory cytokines such as TNF, IL-1, IL-6, IL-23, chemokine ligand (CCL)2, CCL3, CCL5, reactive oxygen and nitrogen species, matrix metalloproteinases (MMP1, -2, -7, -9, and -12), inducible nitric oxide synthetase (iNOS), the FcR types CD16/32/64, and MHCII [32,33,34,35]. In contrast, M2 cells, also called “alternatively activated” macrophages, are induced by T helper cell type 2 (Th2) cytokines such as IL-4 or IL-13 and mainly show anti-inflammatory and regenerative properties such as removal of debris [17,31,32,35]. M2 cells express a broad spectrum of cytokines and enzymes including IL-10, CCL17, CCL22, IL1RII, CD163, scavenger receptors, factor XIII, fibronectin, cyclooxygenase I, and arginase I [32,35,36]. It is known from in vitro studies that TMEV infection itself has an essential impact upon the phenotype of microglia [17]. However, translation of the M1/M2 concept from in vitro to in vivo is discussed controversially, especially since the original M1/M2 data are derived from in vitro research with cultivated macrophages [37]. Future studies integrating genome-wide expression profiles of microglia originating from in vivo studies, such as animal models in combination with computational biology approaches with epigenetic analyses, will provide a more holistic classification of microglia [37,38].

The aims of the present study were to investigate the impact of MMP12 on microglia/astrocyte interaction and to determine the predominant phagocyte type and phenotype of microglia/macrophages in the CNS of wild-type and *Mmp12^−/−^* mice during TME.

## 2. Results

### 2.1. Influence of MMP12 Deficiency on Astrogliosis and Inflammation

**Figure 1:**
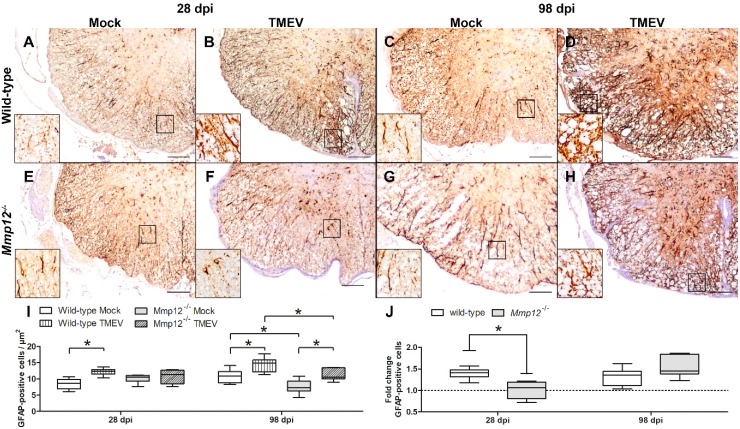
Quantification of astrogliosis by glial fibrillary acidic protein (GFAP) immunohistochemistry in the spinal cord of mock- and Theiler’s murine encephalomyelitis virus (TMEV)-infected wild-type and matrix metalloproteinase 12 knockout (*Mmp12^−/−^*) mice at 28 and 98 days postinfection (dpi; **A**–**H**)**.** Astrocytes show strong cytoplasmic GFAP expression in the gray and white matter. The inserts show higher magnification of the areas delineated by the black rectangles. The bars represent 100 µm in the overview pictures and 35 µm in the inserts. The density of GFAP-positive cells in TMEV-infected wild-type mice was increased at 28 and 98 dpi (**I**) while TMEV-infected *Mmp12^−/−^* mice showed an increased density of GFAP-positive cells at 98 dpi only (**I**). Interestingly, TMEV-infected *Mmp12^−/−^* mice exhibited a reduced density of GFAP-positive cells at 98 dpi compared with wild-type mice and a reduced fold change of GFAP-positive cells in the spinal cord at 28 dpi (**J**) indicating the significant impact of MMP12 on the onset and progression of astrogliosis. Data are shown as box plots with whiskers indicating minimum and maximum values—the lower boundary of the boxes representing the 25th percentile, the upper boundary the 75th; the horizontal line in the boxes represents the median value (**I**,**J**). Significant differences between the groups as detected by the Mann–Whitney *U* test are marked by bars and asterisks (* *p* < 0.05).

Astrocytes of mock- and TMEV-infected animals showed strong cytoplasmic expression of the intermediate filament glial fibrillary acidic protein (GFAP) (Figure 1). TMEV-infected animals showed an increased density of GFAP-positive cells at 28 (wild-type) and 98 days postinfection (dpi; wild-type, *Mmp12^−/−^*) compared with mock-injected control mice. Interestingly, at 28 dpi, *Mmp12^−/−^* mice showed transiently decreased astrogliosis (Figure 1) accompanied by reduced myelitis and demyelination compared with infected wild-type mice at 98 dpi.

**Figure 2:**
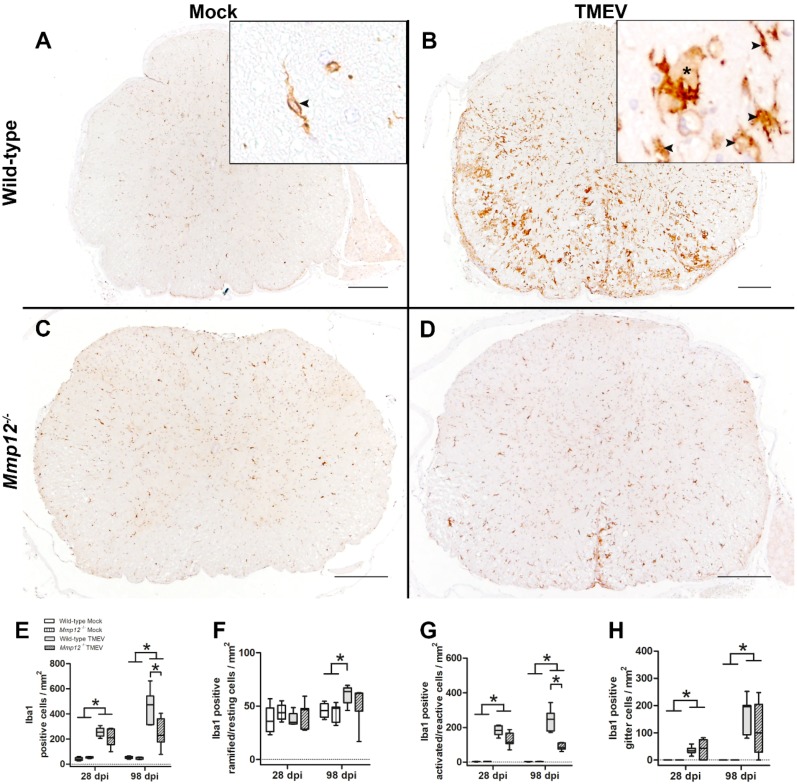
Quantification of microglia/macrophages using immunohistochemistry targeting ionized calcium-binding adaptor molecule 1 (Iba1). Mock-infected wild-type and *Mmp12^−/−^* mice showed low numbers of randomly distributed microglia/macrophages at 28 and 98 dpi (**A**,**C**,**E**). TMEV-infected wild-type and *Mmp12^−/−^* mice showed an increased density of microglia/macrophages, most prominently in the ventral aspect of the white matter, compared with mock-infected mice (**B**,**D**,**E**). The inserts display ramified/resting Iba1 cells (**A**, arrowhead), activated/reactive Iba1 cells (**B**, arrowheads), as well as gitter cells (**B**, asterisk). Bars represent 200 µm in the overview pictures and 25 µm in the inserts. Interestingly, at 98 dpi, the density of microglia/macrophages in TMEV-infected *Mmp12^−/−^* mice was reduced compared with wild-type mice. Furthermore, based on morphology, microglia/macrophage subpopulations, including ramified/resting (**F**), activated/reactive (**G**), as well as gitter cells (**H**), were quantified. The density of ramified/resting microglia remained constant over time in mock-infected as well as TMEV-infected *Mmp12^−/−^* animals. Interestingly *Mmp12^−/−^* mice showed a strong trend (*p* = 0.051) for a reduction in density of activated/reactive microglia/macrophages at 28 dpi and a significant reduction at 98 dpi compared with TMEV-infected wild-type mice. The density of gitter cells was increased in TMEV-infected animals compared with mock-infected animals at both time points, with no significant difference between TMEV-infected groups. Data are shown as box plots with whiskers indicating minimum and maximum values—the lower boundary of the boxes representing the 25th percentile, the upper boundary the 75th; the horizontal line in the boxes represents the median value. Significant differences between the groups as detected by Mann–Whitney *U* test are marked by bars and asterisks (* *p* < 0.05).

Microglia constantly monitor the CNS environment and rapidly respond to triggers such as infection, injury, or stress by changing their morphology and/or their cytokine expression profile [38]. The ability of microglia to transform and reorient their processes towards any site of interest within the CNS is so apparent that it is frequently used as an estimate for microglial activation [37]. Microglia activation preceding astrogliosis has been reported for many diseases [38]. As astrocytes, among other factors, are crucially involved in the activation of microglia/macrophages and vice versa, a reduction/ablation of astrocytes may contribute to reduced microglia activation [39]. Therefore, the next step was to quantify the overall density of microglia/macrophages as well as the density of ramified/resting microglia/macrophages, activated/reactive microglia/macrophages, and gitter cells (Figure 2).

The density of microglia/macrophages in TMEV-infected wild-type and *Mmp12^−/−^* mice was increased at 28 and 98 dpi compared with mock-infected animals (Figure 2). Interestingly, at 98 dpi, the absolute density of microglia/macrophages in TMEV-infected *Mmp12^−/−^* mice was reduced. In addition, TMEV-infected *Mmp12^−/−^* mice showed a strong trend (*p* = 0.051) for a reduced density of activated/reactive microglia/macrophages at 28 dpi and a significantly reduced density at 98 dpi, while the number of ramified/resting microglia/macrophages as well as gitter cells did not show any differences between TMEV-infected groups (Figure 2). These data strongly indicate an indirect (via astrocytes) or direct impact of MMP12 deficiency on microglia activation.

**Figure 3:**
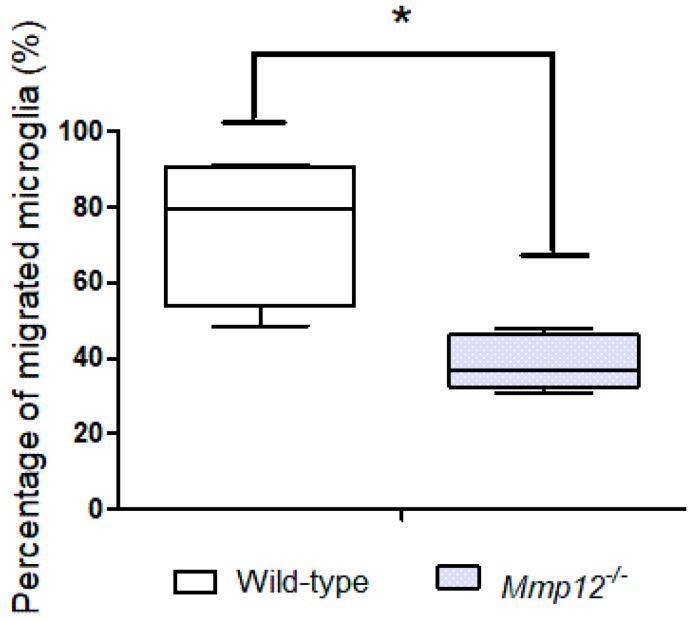
Microglia migration assay revealed a reduced percentage of migrated *Mmp12^−/−^* compared with wild-type microglia cells, indicating an impact of MMP12 on microglia migration capacity. Data are shown as box plots with whiskers indicating minimum and maximum percentage of migrated microglia cells at 6 h postseeding—the lower boundary of the boxes represents the 25th percentile, the upper boundary the 75th; the horizontal line in the boxes represents the median value. Significant differences between groups detected by the Mann–Whitney *U* test are indicated by asterisks (* *p* < 0.05).

To further substantiate this finding, the migratory capacity of microglia was investigated in vitro. At 6 h postseeding, a median of 7952 wild-type microglia reached the lower compartment of the transwell system (minimum 4854; maximum 9123). In contrast, *Mmp12^−/−^* microglia displayed a median of 3668 migrated cells (minimum 3083; maximum 4791). Statistical analysis revealed a reduced number of transmigrated *Mmp12^−/−^* microglia compared with wild-type microglia, indicating an impact of MMP12 on migration of microglia (Figure 3).

**Figure 4:**
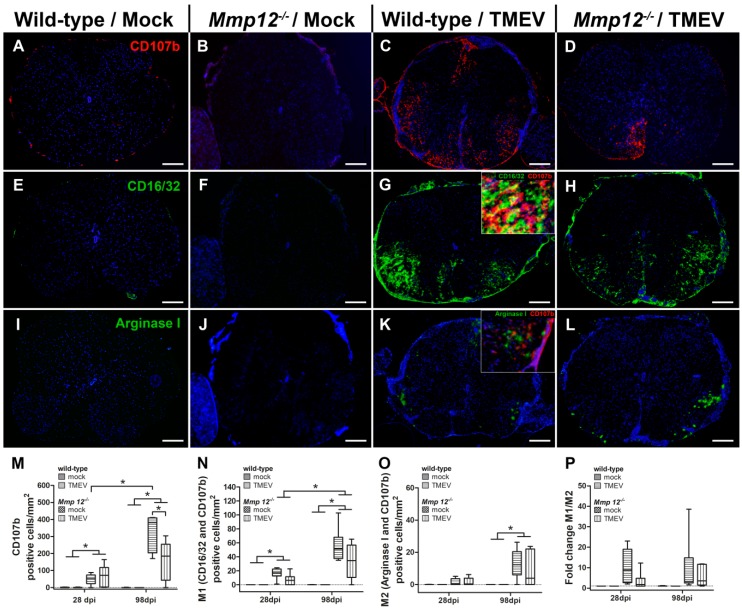
Immunophenotyping of microglia/macrophages in cervical and thoracic spinal cord using immunofluorescence. The density of microglia/macrophages was quantified using CD107b immunofluorescence (**A**–**D**). Mock-infected wild-type (**A**) and *Mmp12^−/−^* (**B**) mice showed only single immunopositive cells compared with TMEV-infected wild-type (**C**) and *Mmp12^−/−^* (**D**) mice at 98 dpi. Wild-type mice showed a significantly increased density of CD107b-positive cells at 98 dpi compared with 28 dpi (**M**). In addition, the density of CD107b-positive cells in TMEV-infected *Mmp12^−/−^* mice was significantly reduced at day 98 compared with TMEV-infected wild-type mice (**M**). M1 cells were identified by immunofluorescence targeting CD16/32 in combination with CD107b (**E**–**H**). Mock-infected wild-type (**E**) and *Mmp12^−/−^* (**F**) mice showed few M1 cells compared with TMEV-infected wild-type (**G**) and *Mmp12^−/−^* (**H**) mice at 98 dpi. The insert (**G**) shows colocalization (yellow color) of CD16/32 (marked in green) and CD107b (marked in red). The density of M1 cells in TMEV-infected mice increased over time (**N**). At 28 dpi, a trend (*p* = 0.073) for a reduced density of M1 cells in TMEV-infected *Mmp12^−/−^* compared with TMEV-infected wild-type mice was detected (**N**). M2 cells were identified by immunofluorescence targeting arginase I in combination with CD107b (**I**–**L**). Mock-infected wild-type (**I**) and *Mmp12^−/−^* (**J**) mice showed low numbers of M2 cells compared with TMEV-infected wild-type (**K**) and TMEV-infected *Mmp12^−/−^* (**L**) mice at 98 dpi. The density of M2 cells was increased in TMEV-infected mice at 98 dpi (**O**). The insert (**K**) shows colocalization (yellow color) of arginase I (marked in green) and CD107b (marked in red). Bars represent 250 µm in the overview pictures and 25 µm in the inserts. Fold change analysis revealed a trend (*p* = 0.073) for M1 dominance in TMEV-infected wild-type mice compared with TMEV-infected *Mmp12^−/−^* mice at 28 dpi (**P**). Data are shown as box plots with whiskers indicating minimum and maximum values—the lower boundary of the boxes representing the 25th percentile, the upper boundary the 75th; the horizontal line in the boxes represents the median value (**M**–**O**). Significant differences between the groups as detected by Mann–Whitney *U* test are marked by bars and asterisks (* *p* < 0.05).

In addition to morphology and migratory capacity, microglia/macrophages were investigated with respect to their phenotype using immunofluorescence targeting CD107b (microglia/macrophages) in combination with CD16/32 (M1 phenotype) and arginase I (M2 phenotype). In accordance with the results of the ionized calcium-binding adaptor molecule 1 (Iba1) immunohistochemistry, mock-infected wild-type and *Mmp12^−/−^* mice showed low numbers of randomly distributed CD107b-positive cells in the spinal cord (Figure 4). TMEV-infected wild-type and *Mmp12^−/−^* mice showed an increased density of CD107b-positive cells compared with their respective mock-infected group at 28 and 98 dpi. In addition, the density of CD107b-positive cells was increased over time in TMEV-infected wild-type mice, while there was no difference in TMEV-infected *Mmp12^−/−^* mice. Interestingly, the density of CD107b-positive cells was reduced in TMEV-infected *Mmp12^−/−^* mice compared with TMEV-infected wild-type mice at 98 dpi. Therefore, the next step was to investigate the contribution of microglia and/or CNS-infiltrating macrophages to the overall number of CD107b-positive cells, with a special focus on the M1 versus the M2 phenotype.

### 2.2. Phenotyping of Microglia/Macrophages in the Spinal Cord

Discrimination between extravasated blood-borne macrophages and microglia in the inflamed spinal cord based on morphology is impossible. This obstacle can be overcome by using immunohistochemistry targeting chemokine receptor 2 (CCR2; [40]). This marker is expressed on blood-borne macrophages but not on microglia [40]. In the present study, quantification of CCR2-labeled cells in the spinal cord revealed only few positive cells in TMEV-infected wild-type and *Mmp12^−/−^* mice. This finding indicates that phagocytes in the spinal cord predominantly consist of microglia.

The distribution of CD16/32- and CD107b-positive cells (M1 cells) in the spinal cord matched the distribution of CD107b-positive cells in TMEV-infected animals (wild-type, *Mmp12^−/−^*). M1 cells were mainly located in the ventral part of the spinal cord and showed a strong cytoplasmic expression of CD16/32 and CD107b. Mock-infected animals showed few M1 cells compared with TMEV-infected wild-type and *Mmp12^−/−^* mice (Figure 4). The density of M1 cells was increased in TMEV-infected animals compared with mock-infected animals at 28 and 98 dpi and increased over time (Figure 4). Furthermore, a trend (*p* = 0.073) towards reduced M1 cell density in TMEV-infected *Mmp12^−/−^* compared with wild-type mice was detected at 28 dpi (Figure 4).

Arginase I- and CD107b-positive cells (M2 cells) were mainly detected in the ventral part of the spinal cord. Mock-infected animals showed only a few positive cells compared with TMEV-infected wild-type and *Mmp12^−/−^* mice (Figure 4). At 98 dpi, the density of M2 cells was increased in TMEV-infected compared with mock-infected wild-type and *Mmp12^−/−^* mice (Figure 4). However, some CD107b-positive cells expressed CD16/32 and arginase I, indicating intermediate stages of microglia/macrophages, possibly underlining their flexibility to switch between phenotypes and presumably contributing to the observed variability of M1/M2 cells in wild-type and *Mmp12^−/−^* mice.

Identifying the predominant phenotype of microglia in TMEV-infected wild-type and *Mmp12^−/−^* mice was performed by calculating the fold change of M1 and M2 cells at 28 and 98 dpi (Figure 4). TMEV-infected groups at all investigated time points showed a fold change larger than 1, indicating the dominance of M1 cells. Although significant differences between TMEV-infected groups were not detected, TMEV-infected *Mmp12^−/−^* mice showed a trend (*p* = 0.073) towards a reduced M1 fold change at 28 dpi (Figure 4).

## 3. Discussion

MMPs are considered to greatly contribute to the pathogenesis of demyelinating diseases such as MS [41,42]. MMP12 is upregulated in MS and its animal model TME, indicating a possible key role of this molecule in the pathogenesis of demyelinating diseases [6,41,43]. In addition, TMEV infection of *Mmp12^−/−^* mice leads to a reduced degree of demyelination, supporting the assumption that MMP12 substantially contributes to demyelination [6]. The aim of the present study was to elucidate the mechanisms contributing to the reduced degree of demyelination in *Mmp12*-deficient mice during TME. Therefore, major focus was given to glia cells with a special emphasis on astrocytes and microglia/macrophages. In this context, in a previous study, an ablation of astrocytes in cuprizone-induced (toxic) demyelination resulted in the insufficient/nonoccurrence of myelin-debris removal and microglia activation [39]. This led to the hypothesis that astrocytes are crucially involved in microglia activation and recruitment. In the present study, a reduced fold change of astrocytes in *Mmp12^−/−^* mice compared with wild-type mice was detected at 28 dpi, while no difference was present at 98 dpi. These observations indicate that the ablation of *Mmp12* may be directly or indirectly responsible for delayed astrocyte proliferation. As astrocytes, especially TMEV-infected astrocytes, are major sources of TNF and IL-12 production [18], delayed astrocyte proliferation may result in reduced activation of microglia and/or have an impact on the phenotype of microglia.

### Contribution of Microglia and Macrophages to TME

Microglia/macrophages are major contributors to inflammation in demyelinating diseases such as MS [35,36,44,45]. However, the quantitative contribution of CNS-infiltrating monocytes to spinal cord inflammation in TME has not been investigated so far. In the present study, CCR2 was applied as a marker for CNS-invading blood-borne macrophages [40]. Low numbers of CCR2-positive cells were detected in the spinal cord of TMEV-infected wild-type and *Mmp12^−/−^* mice at 28 and 98 dpi, indicating that the majority of phagocytes contributing to inflammation represent microglia. Furthermore, investigation of the migratory capacity of *Mmp12^−/−^* and wild-type microglia revealed a reduced migratory capacity of *Mmp12^−/−^* microglia in vitro (Figure 3). Therefore, the reduced number of microglia in *Mmp12^−/−^* mice may be attributed to reduced astrogliosis, facilitating a restricted/insufficient activation of *Mmp12*-deficient microglia and a reduced migratory capacity of *Mmp12^−/−^* microglia or a combination of both [46,47].

Furthermore, the reduced degree of demyelination may result from altered microglial activation and migration, also influencing the M1/M2 balance in TMEV-infected *Mmp12^−/−^* mice. Phagocytes have been classified into proinflammatory, classically activated (M1) cells as well as anti-inflammatory, alternatively activated (M2) cells [35]. M1 cells produce toxic intermediates such as reactive oxygen species as well as reactive nitrogen species, which are necessary to mediate host defense against different pathogens [36]. In contrast, M2 cells are associated with the regulation of tissue repair, wound healing, and debris scavenging [48,49]. In the present study, a dominance of M1 cells (M1/M2 fold change always larger than 1) was present in TMEV-infected wild-type and *Mmp12^−/−^* mice at all investigated time points. Interestingly, a trend towards a reduced density of M1 cells in *Mmp12^−/−^* mice was detected at 28 dpi, while there was no change in the density of arginase I-positive cells at all investigated time points. This was accompanied by reduced astrogliosis at 28 dpi followed by a reduced density of microglia at 98 dpi in TMEV-infected *Mmp12^−/−^* mice compared with TMEV-infected wild-type mice. These data indicate that a lack of MMP12 results in reduced astrogliosis followed by reduced microgliosis, which is mainly attributed to a decreased number of M1 cells but has no impact, however, on the number of M2 cells. Nonetheless, the predominant phenotype of microglia in the spinal cord was still represented by M1 (M1 dominance), indicating a progressive inflammatory disease/milieu in the spinal cord lacking or with insufficient counter-regulatory mechanisms of M2 cells.

## 4. Materials and Methods

SJL.129X-*Mmp12^tm1Sds^* (*Mmp12^−/−^*) and SJL/JOlaHsd (wild-type) mice were used for all experiments [6]. They were housed in isolated ventilated cages (Tecniplast, Hohenpeibenberg, Germany), fed a standard rodent diet (R/M-H; Ssniff Spezialdiäten GmbH, Soest, Germany) ad libitum, and had free access to tap water [6,50,51]. All animal experiments were conducted in accordance with the German law for animal protection and with the European Communities Council Directive 86/609/EEC for the protection of animals used for experimental purposes (Niedersächsisches Landesamt für Verbraucherschutz- und Lebensmittelsicherheit (LAVES), Oldenburg, Germany, permission number: 33.9-42502-04-08/1609).

### 4.1. In Vitro Investigation of Microglia Migration

Microglia were isolated from the neocortex of 5–10, 1–3-day-old *Mmp12^−/−^* and wild-type mice using mechanical dissociation and incubation in calcium-free phosphate-buffered saline (PBS) containing 0.8 g/L of Na-EDTA, 2 mg/mL of trypsin, and 0.2 mg/mL of DNase I (Roche Diagnostics, Mannheim, Germany) as previously described [17,52]. Cells were centrifuged at 250× *g* at 4 °C for 10 min followed by resuspension in Sato’s medium supplemented with 10% fetal calf serum (FCS, Biochrom, Berlin, Germany) and singulation by fire-polished Pasteur pipettes. Thereafter, cells were seeded at a density of 6.6 × 10^5^ cells/cm^2^ in poly-*L*-lysine-coated flasks (Nunc, Wiesbaden, Germany). When cells reached confluency, microglia were isolated by shaking flasks on a rotatory platform (Innova™ 2000; New Brunswick Scientific, New Jersey, NJ, USA) at 37 °C at 150 rpm shaker for 45 min. The enriched microglia cultures displayed a purity of at least 90%, as confirmed by immunostaining targeting Iba1.

The migration ability of wild-type and *Mmp12^−/−^* microglia was assessed by a transwell migration assay. Microglia were seeded on uncoated 24-well Millicell^®^ cell culture inserts with a pore diameter of 8 μm (Merck KGaA, Darmstadt, Germany) at a density of 10,000 cells/well in minimal essential medium Sato’s medium. The lower chamber additionally contained 10% FCS as a chemoattractant. After 6 h of incubation of the transwell systems at 37 °C and 5% CO_2_, migrated cells were scraped, cytospin preparations and Pappenheim staining were performed, and migrated cells were counted.

### 4.2. Virus Infection

Intracerebral infection of 5–6-week-old wild-type and *Mmp12^−/−^* mice with 4.6 × 10^7^ PFU/mouse of the BeAn strain of TMEV or cell culture supernatant (mock) was performed as previously described [6,42,49]. TMEV- and mock-infected mice were killed at 28 (mock-infected: *n* = 6 wild-type, *n* = 6 *Mmp12^−/−^* mice; TMEV-infected: *n* = 6 wild-type, *n* = 7 *Mmp12^−/−^* mice) and 98 (mock-infected: *n* = 6 wild-type, *n* = 7 *Mmp12^−/−^* mice; TMEV-infected: *n* = 7 wild-type, *n* = 7 *Mmp12^−/−^* mice) dpi as previously described [6]. From all animals, segments of cervical and thoracic spinal cord were fixed in 10% formalin or snap frozen in Tissue-Tek^®^ O.C.T.™ Compound (Sakura Finetec Europe B.V., Zoeterwoude, the Netherlands).

### 4.3. Immunohistochemistry

Formalin-fixed, paraffin-embedded 2–3-µm-thick cross sections of cervical and thoracic spinal cord were stained using antibodies targeting Iba1 (polyclonal rabbit, diluted 1:500, Thermo Fisher Scientific, Rockford, Illinois, IL, USA), GFAP (polyclonal rabbit, diluted 1:1000, Dako Diagnostika, Hamburg, Germany), and CCR2 (polyclonal goat, diluted 1:50, Abcam, Cambridge, United Kingdom) followed by a biotinylated secondary antibody (Table 1). Antibody binding was visualized using the avidin–biotin–peroxidase complex method (ABC; Vector Laboratories, Burlingame, CA, USA) followed by 3,3′-diaminobenzidine-tetrahydrochloride and counterstaining with Mayer’s hemalaun. The number of labeled cells in the white matter of the cervical and thoracic spinal cord was counted and calculated as the average density (number of immunolabeled cells in cervical and thoracic spinal cord white matter/white matter area of cervical and thoracic spinal cord). For Iba1, in addition, subpopulations of resting/ramified and activated/reactive microglia as well as gitter cells were counted in the cervical and thoracic spinal cord white matter. Activated/reactive microglia were identified by expanded soma, enlargement, and partial process deramification as previously described [38,53]. Gitter cells were detected as enlarged cells with abundant, vacuolated, and/or foamy cytoplasm. The fold change of GFAP-positive cells was calculated by dividing the number of GFAP-positive cells in the white matter of the cervical and thoracic spinal cord in TMEV-infected mice by the mean number of GFAP-positive cells in the white matter of the cervical and thoracic spinal cord of mock-infected mice.

### 4.4. Immunofluorescence

Methanol-fixed, 3–4-µm-thick frozen sections of cervical and thoracic spinal cord were washed with 0.1% Triton-X-100 in PBS and nonspecific binding was blocked by incubation with 20% goat serum prior to incubation with the respective primary and secondary antibodies (Table 1). Cell nuclei were labeled using bisbenzimide followed by mounting of the slides with fluorescent mounting medium (Dako Diagnostika, Hamburg, Germany). Total numbers of CD107b-positive cells (microglia/macrophages), CD16/32- and CD107b-positive cells (M1 cells), as well as arginase I- and CD107b-positive cells (M2 cells) were counted in the cervical and thoracic spinal cord segments at 28 and 98 dpi. For detecting M1 and M2 cells, consecutive serial sections were used for immunofluorescence labeling targeting CD107b and CD16/32 or arginase I, respectively. CD16/32 is expressed on various cell types, including neutrophils, natural killer cells, and B cells [54]. Therefore, only cells showing a colocalization of CD16/32 and CD107b were counted as M1 cells. Immunofluorescence pictures were superimposed and colocalization of markers was visualized using the negative multiplication function of color layers in Adobe Photoshop™. Fold change for M1 and M2 cells was calculated by dividing the number of M1 cells by the number of M2 cells for each group at each time point.

### 4.5. Statistical Analysis

Statistical analysis was performed using SPSS for Windows (version 17.0; SPSS, Chicago, Illinois, IL, USA). The Kruskal–Wallis test followed by Mann–Whitney *U* post hoc tests were chosen as nonparametric tests for independent samples comparing four or two groups, respectively. Statistical significance was accepted at a *p*-value < 0.05.

## 5. Conclusions

*Mmp12^−/−^* mice showed reduced astrogliosis associated with reduced numbers of activated/reactive microglia. Despite observing a trend towards a reduced M1 phenotype, TMEV-infected *Mmp12^−/−^* mice showed a dominance of proinflammatory M1 microglia in the spinal cord during the demyelinating phase of TME. These data indicate that MMP12 constitutes an important effector molecule involved in microglial migration, activation, and polarization as well as glial scarring.

## Figures and Tables

**Table 1:** Summary of antibodies used for detecting and discriminating astrocytes as well as microglia and/or macrophages.

Primary Antibody	Vendor	Dilution	Secondary Antibody	Vendor
Arginase I	Santa Cruz	1:50	Donkey anti-goat dylight 488	Jackson Immunoresearch
CD107b	AbD Serotec	1:200	Goat anti-rat Cy3	
CD16/32	BD Pharmingen	1:25	Goat anti-rat Cy3	
CCR2	Abcam	1:50	Rabbit anti-goat, biotin	Vector
GFAP	Dako Diagnostika	1:1000	Goat anti-rabbit, biotin	Vector
Iba1	Thermo Fisher Scientific	1:500	Goat anti-rabbit, biotin	Vector

## Abbreviations

CCL	chemokine ligand
CCR2	chemokine receptor 2
CNS	central nervous system
dpi	days postinfection
GFAP	glial fibrillary acidic protein
IFN	interferon
iNOS	inducible nitric oxide synthetase
MMPs	matrix metalloproteinases
*Mmp12^−/−^*	matrix metalloproteinase 12 knockout mice
Th1	T helper cell type 1
Th2	T helper cell type 2
TME	Theiler’s murine encephalomyelitis
TMEV	Theiler’s murine encephalomyelitis virus
TNF	tumor necrosis factor
